# The relationship between poor sleep quality measured by the Pittsburgh Sleep Quality Index and smoking status according to sex and age: an analysis of the 2018 Korean Community Health Survey

**DOI:** 10.4178/epih.e2022022

**Published:** 2022-02-14

**Authors:** Jun Hyun Hwang, Soon-Woo Park

**Affiliations:** Department of Preventive Medicine, Daegu Catholic University School of Medicine, Daegu, Korea

**Keywords:** Sleep quality, Pittsburgh Sleep Quality Index, Smoking behavior, Sex, Age group

## Abstract

**OBJECTIVES:**

Multiple studies have found that cigarette smokers are more likely to experience sleep disturbances than non-smokers. This study aimed to examine various associations between smoking and sleep quality according to sex and age, which have yet to be sufficiently examined in prior studies.

**METHODS:**

Data analysis was conducted using a nationally representative sample of 224,986 Korean adults who participated in the 2018 Korea Community Health Survey. Sleep quality, as the dependent variable, was measured using the Pittsburgh Sleep Quality Index (PSQI), with PSQI scores indicating either good (≤4 points) or poor (>5 points) sleep quality. Multiple logistic regression analysis was performed considering socio-demographic factors, health behaviors, comorbidities, and psychological factors as covariates.

**RESULTS:**

The overall weighted prevalence of poor sleep quality was 39.4% (95% confidence interval, 39.1 to 39.7). In the multivariate model that excluded psychological factors, poor sleep quality positively correlated to smoking for both sexes and all age groups except for male aged ≥65 years. However, in the full model that included psychological factors, statistically significant odds ratios (approximately 1.5) for poor sleep quality according to smoking status were only observed for female under 65 years of age.

**CONCLUSIONS:**

The relationship between poor sleep quality and smoking status differed according to sex and age. In order to improve the quality of sleep, it is necessary to intervene smoking cessation along with solving psychological problems, especially female in middle age and younger.

## GRAPHICAL ABSTRACT

[Fig f2-epih-44-e2022022]

## INTRODUCTION

Despite the lack of definitive consensus in the field of sleep medicine, the term “sleep quality” has been widely used to refer to a collection of sleep-related factors (total sleep time, sleep onset latency, sleep fragmentation, total wake time, sleep efficiency, and sleep disruptive events such as apnea) that are closely related to sleep disorders [1]. In recent years, the number of people who experience sleep disorders has been increasing in several countries. In 2017, approximately one-third of adults in the United States reported sleeping for 6 hours or fewer per day, corresponding to an increase of 15% since 2004 [2]. According to a review article on Australian sleeping habits, frequent (daily or almost daily) sleep disturbances (starting and maintaining sleep, experiencing inadequate sleep), daytime fatigue, drowsiness, and irritability were very prevalent, with incidence rates that ranged from 20% to 35% [3]. In a 2012 survey conducted in the Netherlands, 32.1% of the respondents reported experiencing general sleep disorders, 43.2% reported a lack of sleep, 8.2% experienced insomnia, 5.3% experienced circadian rhythm sleep disorders, 6.1% had parasomnia, 5.9% experienced hypersomnolence, 12.5% experienced restless leg disorder and limb movement during sleep, 7.1% showed sleeprelated breathing disorder, and 12.2% reported 2 or more comorbidities [4].

Several studies have identified smoking as an important factor related to sleep disorders. One meta-analysis found that smokers were 1.47 times more likely to experience sleep-related issues than non-smokers [5]. In a study of young adults, poor sleep quality measured using the Pittsburgh Sleep Quality Index (PSQI) was associated with higher cigarette consumption, more frequent withdrawal symptoms, more frequent cravings, higher modified Fagerstrom Tolerance Questionnaire scores, and meeting more International Classification of Diseases (tenth edition) criteria for tobacco dependence [6,7]. In a study conducted in Indonesia, current smokers and heavy smokers were 1.39 times and 1.91 times more likely to experience sleep disturbances, respectively, than non-smokers, measured using 10 validated indicators of sleep quality and sleep deprivation [8]. Moreover, smokers with obstructive sleep apnea who received a diagnosis on the basis of polysomnography showed a higher apnea-hypopnea index measured by diagnostic criteria, lower mean oxygenation during sleep, and more daytime sleepiness [9].

Low sleep continuity and sleep efficiency in smokers may be partly explained by the effects of nicotine dependence [10]. Due to differences in nicotine metabolism by age and sex and changes in sleep habits with age, the effects of smoking on sleep may also differ according to sex and age [10,11]. Multiple studies have found associations between smoking and sleep disturbance according to sex and age. Among Hispanic/Latino Americans aged 18-76 years, younger female smokers aged 35-54 years were 1.83 times more likely to experience sleep-disordered breathing (SDB), and consumption of 10 cigarettes or more per day was associated with a 2.72-fold increase in the odds of SDB compared to younger female non-smokers. These associations were not observed in male and female participants in other age groups [12]. Stronger associations were observed among female in a Canadian nationwide study, in which elevated urinary cotinine concentrations were found to be associated with a significantly higher likelihood of short or long sleep duration, trouble falling or staying asleep, sleep dissatisfaction, and an increased number of sleep problems [13]. In a study on Japanese workers, the likelihood of difficulty waking up in the morning for male and female smokers was significantly higher than for non-smokers, while increased difficulty initiating sleep and decreased early morning awakening were observed in female smokers only [14]. A study of American adults, however, found no significant interaction between sex and smoking for self-reported snoring, short sleep duration, or poor sleep [15].

To prevent or manage sleep disorders related to smoking, a tailored approach that considers sex and age is essential. However, there have been few studies on this topic, and, to the knowledge of the authors, studies on the relationship between smoking and sleep quality according to both sex and age simultaneously are rare. The purpose of this paper was to investigate the varied relationship between smoking and sleep quality measured using the PSQI according to sex and age in adults using nationally representative data from Korea.

## MATERIALS AND METHODS

### Study population

This study used 2018 Korea Community Health Survey (KCHS) data recorded by the Korea Disease Control and Prevention Agency (formerly the Korea Centers for Disease Control and Prevention) to investigate community health and health-related behaviors. The KCHS was conducted across 255 municipalities that contained community health centers. Participating households were selected using stratified cluster sampling methods; all household members aged ≥ 19 years were included. Approximately 900 individuals per municipality participated in the survey. Qualified interviewers visited the sample households and collected data via face-to-face interviews. Detailed information about the KCHS is available elsewhere [16].

Of the 228,340 participants, data on 224,986 participants were used for the present study after excluding 3,354 participants (1.5% of all participants) with incomplete responses for any of the variables included in this study, including PSQI scores, smoking behaviors, and psychological factors.

### Measures

#### Sleep quality

Sleep quality was measured using the Korean version of the PSQI questionnaire, which has been widely used for evaluating sleep quality [17,18]. The PSQI consists of 19 items measuring 7 components of sleep based on the previous month: (1) subjective sleep quality, (2) sleep latency, (3) sleep duration, (4) sleep efficiency, (5) sleep disturbances, (6) use of sleep medication, and (7) daytime dysfunction. Each component is scored from 0 points to 3 points, and the total PSQI score is calculated as the sum of the 7 components with possible scores ranging from 0 points to 21 points. A PSQI score of > 5 was considered to indicate poor sleep quality in this study.

#### Smoking behaviors

Smoking behaviors were classified into 3 categories based on the amount and frequency of cigarette use: (1) non-smokers, (2) occasional smokers or daily smokers (< 1 pack/day), and (3) heavy daily smokers (≥ 1 pack/day). The proportion of occasional smokers was very low (1.2% overall; 2.3% of males and 0.5% of females), and occasional smokers and daily smokers (< 1 pack/day) were grouped accordingly. People who had never used cigarettes, who had smoked fewer than 100 cigarettes in their lifetime, or who had smoked ≥ 100 cigarettes in their lifetime but quit were classified as non-smokers. The second and third groups, which referred to current smokers, were defined as those who had smoked more than 100 cigarettes in their lifetime and currently smoked. Among current smokers, those who smoked on a non-daily basis (occasional smokers) or smoked less than 1 pack’s worth of cigarettes per day were included in the second group. The third group comprised individuals who smoked more than 1 pack’s worth of cigarettes per day.

#### Other covariates

The socio-demographic characteristics of the study participants were sex (male, female), age (19-35, 36-64, ≥ 65 years, classified based on American Psychological Association criteria) [19], educational level (≤ high school, ≥ college), and employment status (yes, no). The health behavior-related factors included alcohol consumption within 1 year (non-drinker, ≤1 time/mo, 2-4 times/mo, 2-3 times/wk, ≥4 times/wk) and frequency of walking exercise (<4, ≥5 times/wk). Physician-diagnosed hypertension and diabetes were included as possible comorbidities.

Psychological factors, including perceived stress level, depressive mood, and subjective health status, were also included due to their probable association with sleep quality. Perceived stress level was classified into 4 groups (little, some, high, very high) based on the question, “In general, how stressed do you feel in everyday life?” Depressive mood was defined as “yes” or “no” based on the question, “In the past year, have you felt sadness or despair to the extent that you could not complete everyday activities for 2 consecutive weeks or more?” Subjective health status was categorized into 3 groups (high, middle, low).

### Statistical analysis

The weighted percentages of poor sleep quality (PSQI> 5) were calculated in relation to various characteristics. The relationship between sleep quality and smoking was stratified by sex due to the large difference in the prevalence of smoking between male and female in Korea. Logistic regression was used to evaluate the factors associated with sleep quality. Given the strong effect of psychological factors on sleep quality, the results of 2 models (2 types of adjusted odds ratios [aORs]) were presented depending on whether these factors were included. Because of the differences in the metabolic processes related to nicotine and cotinine and in the relationship between sleep quality and tobacco smoking according to sex and age [10,13,20], we performed separate analyses stratified by sex and age group to explore the potential effects on the association between cigarette smoking and sleep quality. An interaction model was used to estimate the effect of smoking on sleep quality according to age.

All analyses were performed using SPSS version 19.0 (IBM Corp., Armonk, NY, USA). A p-value of < 0.05 was considered to indicate statistical significance. Complex SPSS sampling methods were used to accurately represent the Korean adult population.

### Ethics statement

The KCHS data did not include personal information, and participants responded anonymously. The raw KCHS data are publicly available and were thus exempted from a review by an Institutional Review Board by the Korea Disease Control and Prevention Agency.

## RESULTS

The characteristics of the study participants and the corresponding prevalence rates of poor sleep quality are shown in Table 1. Among the study participants, 39.4% had poor sleep quality. Sleep quality was lower among female, elderly participants, those with a low education level, those who were unemployed, those who participated in walking exercise less often, and those who had been diagnosed with hypertension or diabetes. In particular, the difference in sleep quality according to psychological factors was meaningfully large (gap between the maximum and the minimum of > 20%p) for each factor. However, sleep quality showed no consistent dose-response relationship according to the smoking or drinking amount when the results were not stratified by sex (Table 1).

Table 2 shows the factors related to poor sleep quality after stratification by sex. The odds ratio (OR) for poor sleep quality compared to non-smokers was 1.09 (95% confidence interval [CI], 1.05 to 1.14) for male occasional and daily smokers (< 1 pack/day) and 1.30 (95% CI, 1.24 to 1.36) for male daily smokers (≥ 1 pack/day), while it was 1.96 (95% CI, 1.80 to 2.15) for female occasional and daily smokers (< 1 pack/day) and 2.45 (95% CI, 1.97 to 3.04) for female daily smokers (≥ 1 pack/day). In the multivariate model in which psychological factors were excluded, sleep quality and smoking showed a significant dose-response relationship as in the univariate model. In the model that was further adjusted for psychological factors, the strength of the relationship between smoking and sleep quality was weaker than that of the previous model, and no dose-response relationship was observed, but the results were still significant for female (aOR, 1.50; 95% CI, 1.36 to 1.65 for occasional and daily smokers [< 1 pack/day]; aOR, 1.45; 95% CI, 1.16 to 1.81 for daily smokers [≥ 1 pack/day]). Among males, only those who smoked more than 1 pack per day showed significantly lower sleep quality, with a decreased aOR of 1.08 (95% CI, 1.02 to 1.14). In this fully adjusted model, all variables except for comorbidities correlated with poor sleep quality among both sexes, and the aOR of psychological factors was the largest (Table 2).

In each group classified by sex and age, the aORs without adjustment for psychological factors related to poor sleep quality increased with a higher frequency/amount of smoking for both male and female aged 19-35 years, and the strength of this association was smaller for those aged 36-64 years and male compared to those aged 19-35 years and female. After additional adjustment for psychological factors, a significant association between smoking and sleep quality persisted for female aged 19-35 years (aOR, 1.49; 95% CI, 1.24 to 1.79 for occasional and daily smokers [< 1 pack/day]) and 36-64 years (aOR, 1.56; 95% CI, 1.37 to 1.77 for occasional and daily smokers [< 1 pack/day]; aOR, 1.49; 95% CI, 1.14 to 1.95 for daily smokers [≥ 1 pack/day]), but no dose-response relationship according to smoking level was observed, and the results for male and female over 65 years of age were not statistically significant (Table 3). Similarly, as a result of the analysis by age, in which the participants were stratified across 10-year age groups, a relationship between smoking and sleep quality in the final model was not clearly observed in male but showed a significant association in female under 70 years of age (Supplementary Material 1). In addition, when classifying non-smokers as neversmokers and past smokers, a significant association was observed among those under 65 years of age and in male, but the strength of the association was lower than that of females (Supplementary Material 2).

**Figure 1.**
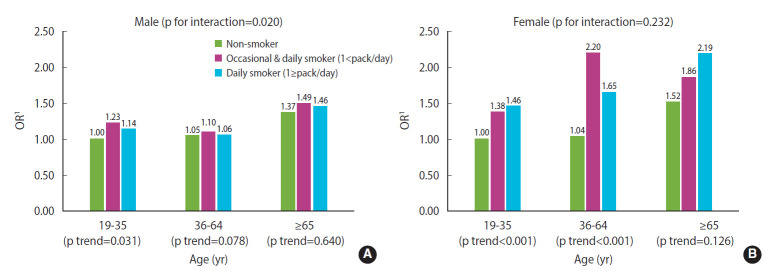
Adjusted odds ratios (ORs) for poor sleep quality by smoking frequency/amount and age group (A: male, B: female). ^1^Adjusted for frequency of age, education level, employment, alcohol consumption, walking exercise, history of hypertension or diabetes, perceived stress level, depressive mood, and subjective health status.

The results from the interaction model are shown in Figure 1. In males, a significant association between smoking and poor sleep quality was only found among those aged younger than 35, indicating that there was a significant interaction between age and smoking (p for interaction=0.020). On the contrary, for females, smoking tended to affect sleep quality regardless of age, so there was no interaction effect between the 2 factors (p for interaction=0.232) (Figure 1).

## DISCUSSION

The overall weighted prevalence of poor sleep quality in this study was 39.4% (95% CI, 39.1 to 39.7), which is similar to the 36% prevalence rate found among the German general population [21] and much higher than the 26.6% prevalence rate reported in China [22]. The high prevalence of poor sleep quality highlights a need for further research to elucidate the causes of sleep disorders and other related factors.

In this study, there was a significant relationship between poor sleep quality and smoking only in females under the age of 65, while no significant results were found for males and elderly females. In other words, the relationship between smoking and sleep quality differed according to sex and age, which was also supported by the interaction model. These were derived by applying various modeling methods. When the results were not adjusted for confounding variables, statistically significant correlations between poor sleep quality and smoking status, as well as dose-response relationships, were observed in both sexes. Even after adjustment for socioeconomic factors such as age, education level, and employment status; health behaviors such as alcohol consumption and walking exercise; and comorbidities such as hypertension and diabetes, these correlations showed a similar pattern. These results are consistent with the findings of previous studies that observed associations between smoking and sleep quality measured using the PSQI among the general population [23-25]. The aforementioned confounders were all significantly associated with poor sleep quality in this study. Nevertheless, the aORs remained almost unchanged compared to the ORs, which suggests that smoking was associated with sleep independent of these factors. However, when stratified by age, a distinct dose-response relationship between poor sleep quality and smoking status was observed only for participants aged 19-35 years. When psychological factors, including perceived stress level, depressive mood, and subjective health level, were adjusted further, the ORs for poor sleep quality decreased generally, and statistically significant ORs for poor sleep quality according to smoking status were observed only in female below 65 years of age. Previous studies that found a significant relationship between smoking and sleep quality did not include elderly participants [23] or included only a small proportion of elderly participants [24], and did not stratify the results by age group [23,25]. Moreover, some of these studies did not consider psychological factors in their statistical models [26,27]. This study attempted a different approach to illustrate the relationship between smoking and sleep quality by adjusting for various potential confounders, including psychological factors, and by considering the interaction between sex and age group, which was not considered in previous studies.

The mechanisms through which cigarette smoking causes sleep disturbances have been studied by several researchers. Nicotine, which is the major pharmacologically active compound in tobacco, affects the central nervous system by stimulating the nicotine-acetylcholine receptor. The activation of nicotinic receptors leads to the release of several neurotransmitters, including acetylcholine, dopamine, serotonin, norepinephrine, and gamma-aminobutyric acid. The deleterious effects of nicotine on sleep quality may be attributable to the independent and interactive effects of these neurotransmitters on the central mechanisms that regulate the sleep-wake cycle, resulting in increased sleep latency and contributing to sleep disturbance [28-30]. In addition to the direct action of nicotine on sleep mechanisms, smokers may experience withdrawal symptoms and cravings due to a reduction in their nicotine levels after sleep since the half-life of nicotine in the human body is only 1 hour to 2 hours. This may reduce smokers’ quality of sleep [8,31]. Medical conditions and lifestyle habits related to smoking may lead to a decrease in the quality of sleep. Compared to non-smokers, smokers are more likely to experience mental health problems such as stress and depression as well as physical health problems such as cardiovascular and respiratory diseases, including obstructive lung disease, diabetes, and obesity. Smokers also tend to consume more alcohol and caffeine than non-smokers [8,30,32,33].

Poor sleep is known to be associated with poor health-related quality of life and symptoms of depression and anxiety, all of which are associated with active cigarette smoking [8]. Mental problems, such as depression, stress, and anxiety, have been consistently found to strongly relate to smoking in most studies, although findings related to their causal relationships have been inconsistent. Self-rated health has also been reported to be associated with smoking [26,27,34]. In this study, perceived stress level, depressive mood, and subjective health level showed distinct dose-response relationships or statistically significant independent relationships with poor sleep quality in individuals of both sexes. Thus, these variables showed a greater correlation with poor sleep quality than smoking when the correlation between smoking and poor sleep quality decreased or when statistical significance was no longer observed after adjusting for them. However, since the direction of the relationship between these variables and smoking is unclear, the potential effect of smoking on poor sleep quality should not be underestimated.

When stratified by sex and age, the ORs (crude and aORs for all characteristics except for psychological factors) for poor sleep quality according to smoking status tended to decrease as age increased for both sexes. This is presumed to have been partly due to differences in nicotine metabolism according to age. In a study that included healthy elderly and adult participants, compared to adults aged 22-43 years, elderly individuals aged 65-76 years showed significantly lower total nicotine clearance (-23%), nonrenal clearance (-21%), and renal clearance (-49%). The maximal heart rate response to nicotine also decreased for elderly participants (-29%). This decrease in nicotine clearance may delay or alleviate withdrawal symptoms or cravings during sleep [20]. In addition, the decrease in the relationship between smoking and sleep quality with age can also be explained by natural changes in sleep patterns as a part of the normal aging process, which may reduce the relative effects of smoking on sleep quality [35]. One study found that there was a significantly longer wake time after the onset of sleep, reduction in total sleep time and sleep efficiency, and higher arousal index, which were measured using polysomnography, among elderly male and female after adjusting for race, the use of hormone replacement therapy, smoking history, sleep apnea, and chronic health conditions [36]. The lack of a dose-response relationship between poor sleep quality and smoking status in individuals older than 35 years is also presumed to be caused by the relative decrease in the effect of smoking on sleep quality with age. However, further research is needed to explain these findings.

Another noteworthy point in this study is that the ORs for poor sleep quality related to smoking status in female were greater than those for male in all age groups. Differences by sex concerning the sensitivity and metabolization rate of nicotine may explain the stronger association between tobacco smoke exposure and poorer sleep quality among female [13]. In a study that included twins as the subjects, the clearance of nicotine and cotinine, the nicotine-to-cotinine clearance ratio, and the ratio of *trans-3*′-hydroxycotinine to cotinine were significantly higher in female than in male. However, the findings concerning menopausal or postmenopausal female did not differ from those concerning male. Thus, accelerated nicotine metabolism appears to be influenced by sex hormones, specifically estrogen [37]. In addition, menopause is a strong risk factor for SDB, and the risk of SDB for male is 2-3 times higher than for female. Therefore, the effects of smoking may be less powerful for menopausal female and male [12]. This could partly explain why the ORs for female aged 65 years and older were lower than those for younger female, along with the other aforementioned effects of aging.

This study had several limitations. First, the causal relationship between smoking behaviors and sleep quality could not be determined, which is an inherent limitation of any cross-sectional survey. Second, occasional smokers and daily smokers who smoked fewer than 1 pack of cigarettes per day were not subdivided into separate groups because of the very small number of occasional smokers. Third, the failure to account for confounders known to affect sleep quality, such as obesity and respiratory disease, may have also influenced the results [38]. Although the results were not shared, an analysis that included obesity was also attempted in this study. However, since height and weight information were omitted in more than 20% of cases and obesity was not related to sleep quality, these data were excluded from the final analysis. Fourth, past smokers and never-smokers were not distinguished in the main analysis. However, the mechanism that causes poor sleep quality in smokers may be explained by nicotine metabolism or withdrawal symptoms experienced by current smokers. Therefore, never-smokers and past smokers can be classified into the same group since they are both likely not significantly affected by nicotine metabolism. The analysis results for non-smokers after distinguishing between past smokers and never-smokers are presented in Supplementary Materials 2, and these results were consistent with the main findings (the relationship between smoking and sleep quality was prominent in female, especially those younger than middle age). Fifth, this study has limitations with regard to the validity and accuracy of the participants’ responses. However, the KCHS was conducted using a standardized interviewing method by well-trained interviewers, and the questionnaires used to measure depressive mood, perceived stress, and subjective health status have been widely used in many previous studies. Therefore, the limitations related to validity and accuracy are unlikely to have been influential.

One of the major strengths of this study was that various socio-demographic factors, health behaviors, comorbidities, and psychological factors, all of which may simultaneously affect smoking status and sleep quality, were included as confounding factors. The primary strength of this study may be that the relationship between sleep quality evaluated using the PSQI and smoking was analyzed while also considering the interaction between sex and age among a large, nationally representative sample. Although some studies have analyzed sleep disorders according to sex or age, few studies have considered sex and age simultaneously, especially using the PSQI. This study on the relationship between PSQI scores and smoking according to sex and age could provide evidence suggesting the need to provide various services or programs for those who suffer from sleep disorders.

The results of this study suggest that interventions for sleep disorders should specifically include counseling and treatment for smoking cessation targeted to female, especially females who are in middle age or younger. These interventions should be conducted alongside measures to treat psychological problems such as depression and stress.

## Figures and Tables

**Figure f2-epih-44-e2022022:**
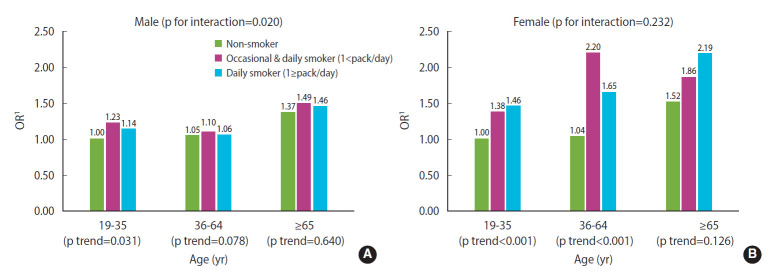


**Table 1.** Characteristics of the participants and poor sleep quality (PSQI>5)

Characteristics			Study population		Poor sleep quality
			Unweighted n	Proportion (weighted %)	Weighted % (95% CI)
Total			224,986	100.0	39.4 (39.1, 39.7)
Smoking frequency/amount					
	Non-smoker		185,975	79.8	39.6 (39.3, 40.0)
	Occasional smoker or daily smoker (<1 pack/day)		23,483	13.0	37.8 (37.0, 38.5)
	Daily smoker (≥1 pack/day)		15,528	7.2	39.8 (38.8, 40.8)
Socio-demographic factors					
	Sex				
		Male	100,914	49.6	34.2 (33.8, 34.6)
		Female	124,072	50.4	44.5 (44.2, 44.9)
	Age (yr)				
		19-35	37,947	26.6	35.2 (34.7, 35.8)
		36-64	116,347	55.2	38.1 (37.7, 38.4)
		≥65	70,692	18.2	49.5 (49.0, 50.1)
	Education level				
		≤High school	156,702	58.4	42.8 (42.5, 43.2)
		≥College	68,284	41.6	34.6 (34.1, 35.0)
	Employment status				
		No	86,075	36.4	45.0 (44.5, 45.4)
		Yes	138,911	63.6	36.2 (35.9, 36.6)
Health behavior factors					
	Alcohol consumption within 1 yr				
		Non-drinker	72,826	24.3	43.2 (42.7, 43.8)
		≤1 time/mo	57,843	27.7	39.4 (38.8, 39.9)
		2-4 times/mo	44,898	24.4	35.9 (35.4, 36.5)
		2-3 times/wk	31,144	16.3	37.8 (37.1, 38.5)
		≥4 times/wk	18,275	7.4	41.9 (41.0, 42.9)
	Walking exercise (times/wk)				
		<4	124,913	50.2	41.2 (40.8, 41.6)
		≥5	100,073	49.8	37.6 (37.2, 38.0)
Comorbidities					
	Hypertension				
		No	161,054	79.6	37.5 (37.2, 37.8)
		Yes	63,932	20.4	46.9 (46.4, 47.5)
	Diabetes				
		No	200,089	91.8	38.6 (38.3, 38.9)
		Yes	24,897	8.2	49.0 (48.2, 49.9)
Psychological factors					
	Perceived stress level				
		Little	55,127	19.4	29.9 (29.4, 30.5)
		Some	117,334	54.8	35.2 (34.8, 35.5)
		High	44,854	22.0	53.4 (52.8, 54.0)
		Very high	7,671	3.7	67.9 (66.6, 69.2)
	Depressive mood				
		No	212,060	94.1	37.3 (37.0, 37.6)
		Yes	12,926	5.9	73.5 (72.5, 74.5)
	Subjective health status				
		High	79,265	39.5	27.5 (27.1, 27.9)
		Middle	100,026	45.9	42.4 (42.0, 42.8)
		Low	45,695	14.5	62.1 (61.5, 62.7)

PSQI, Pittsburgh Sleep Quality Index; CI, confidence interval.

**Table 2.** Unadjusted and adjusted ORs for poor sleep quality (PSQI>5) using multiple logistic regression

Variables		Subgroup	Male			Female		
			Crude OR (95% CI)	Adjusted OR (95% CI)^1^	Adjusted OR (95% CI)^2^	Crude OR (95% CI)	Adjusted OR (95% CI)^1^	Adjusted OR (95% CI)^2^
Smoking frequency/amount		Non-smoker	1.00 (reference)	1.00 (reference)	1.00 (reference)	1.00 (reference)	1.00 (reference)	1.00 (reference)
		Occasional smoker and daily smoker (<1 pack/day)	1.09 (1.05, 1.14)^*^	1.14 (1.09, 1.19)^*^	1.04 (0.99, 1.09)	1.96 (1.80, 2.15)^*^	1.92 (1.75, 2.11)^*^	1.50 (1.36, 1.65)^*^
		Daily smoker (≥1 pack/day)	1.30 (1.24, 1.36)^*^	1.27 (1.21, 1.34)^*^	1.08 (1.02, 1.14)^*^	2.45 (1.97, 3.04)^*^	2.26 (1.82, 2.82)^*^	1.45 (1.16, 1.81)^*^
Socio-demographic factors								
	Age (yr)	19-35	1.00 (reference)	1.00 (reference)	1.00 (reference)	1.00 (reference)	1.00 (reference)	1.00 (reference)
		36-64	1.14 (1.09, 1.19)^*^	1.08 (1.04, 1.13)^*^	1.01 (0.96, 1.06)	1.11 (1.07, 1.15)^*^	1.04 (1.00, 1.08)	1.04 (1.00, 1.09)
		≥ 65	1.47 (1.40, 1.54)^*^	1.22 (1.15, 1.30)^*^	1.28 (1.20, 1.36)^*^	2.02 (1.94, 2.11)^*^	1.52 (1.44, 1.60)^*^	1.51 (1.43, 1.61)^*^
	Education level	≤ High school	1.24 (1.20, 1.28)^*^	1.11 (1.07, 1.15)^*^	1.12 (1.08, 1.16)^*^	1.53 (1.49, 1.59)^*^	1.23 (1.19, 1.28)^*^	1.20 (1.16, 1.25)^*^
		≥ College	1.00 (reference)	1.00 (reference)	1.00 (reference)	1.00 (reference)	1.00 (reference)	1.00 (reference)
	Employment status	No	1.23 (1.18, 1.28)^*^	1.20 (1.14, 1.25)^*^	1.13 (1.08, 1.18)^*^	1.37 (1.33, 1.41)^*^	1.18 (1.14, 1.22)^*^	1.20 (1.16, 1.24)^*^
		Yes	R1.00 (reference)	1.00 (reference)	1.00 (reference)	1.00 (reference)	1.00 (reference)	1.00 (reference)
Health behavior factors								
	Alcohol consumption within 1 yr	Non-drinker	1.00 (reference)	1.00 (reference)	1.00 (reference)	1.00 (reference)	1.00 (reference)	1.00 (reference)
		≤1 time/mo	0.95 (0.90, 1.00)^*^	1.05 (1.00, 1.11)	1.11 (1.05, 1.18)^*^	0.83 (0.80, 0.86)^*^	1.03 (0.99, 1.07)	1.05 (1.01, 1.10)^*^
		2-4 times/mo	0.84 (0.80, 0.89)^*^	0.96 (0.91, 1.02)	1.06 (1.00, 1.12)	0.81 (0.78, 0.85)^*^	1.09 (1.04, 1.14)^*^	1.15 (1.10, 1.21)^*^
		2-3 times/wk	0.99 (0.94, 1.05)	1.10 (1.04, 1.17)^*^	1.20 (1.13, 1.27)^*^	0.92 (0.87, 0.97)^*^	1.20 (1.13, 1.27)^*^	1.22 (1.15, 1.30)^*^
		≥4 times/wk	1.23 (1.16, 1.30)^*^	1.25 (1.17, 1.32)^*^	1.28 (1.20, 1.36)^*^	1.27 (1.15, 1.41)^*^	1.43 (1.29, 1.59)^*^	1.39 (1.24, 1.55)^*^
	Walking exercise (times/wk)	<4	1.14 (1.11, 1.18)^*^	1.13 (1.10, 1.17)^*^	1.05 (1.01, 1.09)^*^	1.17 (1.14, 1.21)^*^	1.15 (1.12, 1.19)^*^	1.06 (1.03, 1.10)^*^
		≥5	1.00 (reference)	1.00 (reference)	1.00 (reference)	1.00 (reference)	1.00 (reference)	1.00 (reference)
Comorbidities								
	Hypertension	No	1.00 (reference)	1.00 (reference)	1.00 (reference)	1.00 (reference)	1.00 (reference)	1.00 (reference)
		Yes	1.30 (1.25, 1.35)^*^	1.13 (1.08, 1.18)^*^	1.01 (0.97, 1.06)	1.71 (1.65, 1.77)^*^	1.24 (1.19, 1.29)^*^	1.09 (1.04, 1.13)^*^
	Diabetes	No	1.00 (reference)	1.00 (reference)	1.00 (reference)	1.00 (reference)	1.00 (reference)	1.00 (reference)
		Yes	1.40 (1.33, 1.48)^*^	1.21 (1.14, 1.28)^*^	1.01 (0.96, 1.08)	1.79 (1.71, 1.89)^*^	1.28 (1.21, 1.35)^*^	1.06 (1.00, 1.12)
Psychological factors								
	Perceived stress level	Little	1.00 (reference)	-	1.00 (reference)	1.00 (reference)	-	1.00 (reference)
		Some	1.30 (1.24, 1.36)^*^	-	1.39 (1.32, 1.46)^*^	1.24 (1.20, 1.29)^*^	-	1.43 (1.37, 1.49)^*^
		High	2.58 (2.45, 2.72)^*^	-	2.45 (2.31, 2.60)^*^	2.83 (2.70, 2.96)^*^	-	2.78 (2.64, 2.91)^*^
		Very high	5.03 (4.57, 5.54)^*^	-	3.80 (3.42, 4.21)^*^	5.11 (4.67, 5.60)^*^	-	3.78 (3.41, 4.18)^*^
	Depressive mood	No	1.00 (reference)	-	1.00 (reference)	1.00 (reference)	-	1.00 (reference)
		Yes	4.90 (4.49, 5.35)^*^	-	2.89 (2.64, 3.17)^*^	4.19 (3.92, 4.47)^*^	-	2.45 (2.29, 2.63)^*^
	Subjective health status	High	1.00 (reference)	-	1.00 (reference)	1.00 (reference)	-	1.00 (reference)
		Middle	1.83 (1.77, 1.90)^*^	-	1.66 (1.59, 1.72)^*^	1.97 (1.91, 2.04)^*^	-	1.70 (1.64, 1.76)^*^
		Low	3.62 (3.43, 3.81)^*^	-	2.64 (2.49, 2.79)^*^	4.67 (4.47, 4.84)^*^	-	2.93 (2.79, 3.09)^*^

PSQI, Pittsburgh Sleep Quality Index; OR, odds ratio; CI, confidence interval.

1 Adjusted for frequency of age, education level, employment status, alcohol consumption, walking exercise, and history of hypertension or diabetes.

2 Additionally adjusted for perceived stress level, depressive mood, and subjective health status to model 1.

* p<0.05.

**Table 3.** Unadjusted and adjusted ORs for poor sleep quality (PSQI>5) according to smoking status by sex and age

Variables			Male			Female		
			Crude OR (95% CI)	Adjusted OR (95% CI)^1^	Adjusted OR (95% CI)^2^	Crude OR (95% CI)	Adjusted OR (95% CI)^1^	Adjusted OR (95% CI)^2^
Age (yr)								
	19-35							
		Non-smoker	1.00 (reference)	1.00 (reference)	1.00 (reference)	1.00 (reference)	1.00 (reference)	1.00 (reference)
		Occasional smoker or daily smoker (<1 pack/day)	1.21 (1.11, 1.31)^*^	1.15 (1.06, 1.26)^*^	1.07 (0.97, 1.16)	2.12 (1.80, 2.50)^*^	2.00 (1.68, 2.37)^*^	1.49 (1.24, 1.79)^*^
		Daily smoker (≥1 pack/day)	1.59 (1.41, 1.80)^*^	1.43 (1.26, 1.62)^*^	1.15 (1.00, 1.32)	3.43 (2.12, 5.55)^*^	3.24 (2.00, 5.25)^*^	1.53 (0.93, 2.50)
	36-64							
		Non-smoker	1.00 (reference)	1.00 (reference)	1.00 (reference)	1.00 (reference)	1.00 (reference)	1.00 (reference)
		Occasional smoker or daily smoker (<1 pack/day)	1.12 (1.06, 1.19)^*^	1.11 (1.05, 1.18)^*^	1.01 (0.96, 1.08)	2.24 (1.99, 2.53)^*^	1.98 (1.75, 2.23)^*^	1.56 (1.37, 1.77)^*^
		Daily smoker (≥1 pack/day)	1.33 (1.25, 1.41)^*^	1.23 (1.16, 1.31)^*^	1.06 (1.00, 1.13)	2.52 (1.93, 3.30)^*^	2.07 (1.58, 2.70)^*^	1.49 (1.14, 1.95)^*^
	≥65							
		Non-smoker	1.00 (reference)	1.00 (reference)	1.00 (reference)	1.00 (reference)	1.00 (reference)	1.00 (reference)
		Occasional smoker or daily smoker (<1 pack/day)	1.11 (1.00, 1.22)^*^	1.08 (0.98, 1.19)	1.02 (0.92, 1.13)	1.56 (1.22, 1.98)^*^	1.53 (1.21, 1.94)^*^	1.27 (0.99, 1.62)
		Daily smoker (≥1 pack/day)	1.01 (0.89, 1.15)	1.02 (0.90, 1.16)	0.94 (0.82, 1.08)	1.52 (0.93, 2.49)	1.52 (0.93, 2.50)	1.06 (0.60, 1.87)

PSQI, Pittsburgh Sleep Quality Index; OR, odds ratio; CI, confidence interval.

1 Adjusted for frequency of age, education level, employment status, alcohol consumption, walking exercise, and history of hypertension or diabetes.

2 Additionally adjusted for perceived stress level, depressive mood, and subjective health status to model 1.

* p<0.05.

## SUPPLEMENTARY MATERIALS

Supplementary Material 1. Unadjusted and adjusted odds ratios for poor sleep quality (PSQI > 5) according to smoking status by sex and age group (by 10 year group)

Supplementary Material 2 Unadjusted and adjusted odds ratios for poor sleep quality (PSQI > 5) according to smoking status (non-smoker subdivided into never-smoker and past smoker) by sex and age group
