# Supplementary material for: The relationship between poor sleep quality measured by the Pittsburgh Sleep Quality Index and smoking status according to sex and age: an analysis of the 2018 Korean Community Health Survey
Source: Epidemiol Health. 2022 Feb 14;44:e2022022. doi: 10.4178/epih.e2022022 (PMC9117098; doi:10.4178/epih.e2022022)
Supplement: Supplementary Material 1. — Unadjusted and adjusted odds ratios for poor sleep quality (PSQI > 5) according to smoking status by sex and age group (by 10 year group) [file epih-44-e2022022-suppl1.docx]

Supplementary Material 1. Unadjusted and adjusted odds ratios for poor sleep quality (PSQI > 5) according to smoking status by sex and age group (by 10 year group)

| Category | Subgroup | Male | | |  | Female | | |
| --- | --- | --- | --- | --- | --- | --- | --- | --- |
| Age |  | Crude OR  (95% CI) | Adjusted OR^1^  (95% CI) | Adjusted OR^2^  (95% CI) |  | Crude OR  (95% CI) | Adjusted OR^1^  (95% CI) | Adjusted OR^2^  (95% CI) |
| 19-29 | Non-smoker | Reference | Reference | Reference |  | Reference | Reference | Reference |
|  | Occasional smoker & Daily smoker (<1 pack per day) | 1.23  (1.11-1.37)* | 1.18  (1.06-1.32)* | 1.09  (0.97-1.23) |  | 2.42  (1.98-2.96)* | 2.21  (1.80-2.72)* | 1.57  (1.25-1.97)* |
|  | Daily smoker (≥1 pack per day) | 1.79  (1.52-2.11)* | 1.61  (1.36-1.91)* | 1.28  (1.06-1.54)* |  | 3.43  (1.77-6.68)* | 3.09  (1.59-6.03)* | 1.70  (0.87-3.32) |
| 30-39 | Non-smoker | Reference | Reference | Reference |  | Reference | Reference | Reference |
|  | Occasional smoker & Daily smoker (<1 pack per day) | 1.17  (1.07-1.28)* | 1.11  (1.01-1.22)* | 1.05  (0.95-1.16) |  | 2.20  (1.77-2.73)* | 2.02  (1.61-2.52)* | 1.65  (1.32-2.06)* |
|  | Daily smoker (≥1 pack per day) | 1.35  (1.19-1.53)* | 1.20  (1.06-1.37)* | 1.03  (0.89-1.18) |  | 3.23  (1.96-5.34)* | 2.76  (1.67-4.57)* | 1.36  (0.81-2.29) |
| 40-49 | Non-smoker | Reference | Reference | Reference |  | Reference | Reference | Reference |
|  | Occasional smoker & Daily smoker (<1 pack per day) | 1.16  (1.06-1.27)* | 1.13  (1.03-1.24)* | 1.04  (0.94-1.14) |  | 2.17  (1.79-2.61)* | 1.79  (1.48-2.17)* | 1.47  (1.19-1.82)* |
|  | Daily smoker (≥1 pack per day) | 1.41  (1.28-1.56)* | 1.29  (1.16-1.42)* | 1.10  (0.99-1.22) |  | 3.91  (2.43-6.30)* | 3.15  (1.94-5.10)* | 2.10  (1.30-3.40)* |
| 50-59 | Non-smoker | Reference | Reference | Reference |  | Reference | Reference | Reference |
|  | Occasional smoker & Daily smoker (<1 pack per day) | 1.10  (1.01-1.22)* | 1.06  (0.96-1.17) | 0.99  (0.89-1.09) |  | 2.23  (1.82-2.73)* | 2.02  (1.65-2.49)* | 1.57  (1.25-1.96)* |
|  | Daily smoker (≥1 pack per day) | 1.27  (1.16-1.39)* | 1.17  (1.06-1.29)* | 1.05  (0.95-1.16) |  | 1.81  (1.20-2.72)* | 1.49  (0.99-2.24) | 1.16  (0.77-1.75) |
| 60-69 | Non-smoker | Reference | Reference | Reference |  | Reference | Reference | Reference |
|  | Occasional smoker & Daily smoker (<1 pack per day) | 1.05  (0.93-1.17) | 1.02  (0.91-1.14) | 0.93  (0.82-1.04) |  | 2.04  (1.59-2.63)* | 1.94  (1.51-2.50)* | 1.54  (1.19-2.00)* |
|  | Daily smoker (≥1 pack per day) | 1.19  (1.06-1.34)* | 1.11  (0.99-1.26) | 0.99  (0.87-1.12) |  | 1.86  (1.11-3.11)* | 1.81  (1.08-3.05)* | 1.24  (0.69-2.22) |
| ≥70 | Non-smoker | Reference | Reference | Reference |  | Reference | Reference | Reference |
|  | Occasional smoker & Daily smoker (<1 pack per day) | 1.10  (0.97-1.24) | 1.07  (0.95-1.21) | 1.04  (0.91-1.18) |  | 1.36  (1.01-1.83)* | 1.35  (1.01-1.80)* | 1.12  (0.83-1.52) |
|  | Daily smoker (≥1 pack per day) | 1.04  (0.87-1.25) | 1.03  (0.86-1.24) | 1.00  (0.82-1.22) |  | 1.46  (0.75-2.85) | 1.44  (0.73-2.81) | 1.09  (0.52-2.29) |

OR, odds ratio; CI, confidence interval

*p < 0.05

^1^ Adjusted for frequency of age, education level, employment, alcohol consumption, walking exercise, and history of hypertension or diabetes.

^2^ Additionally adjusted for perceived stress level, depressive mood, and subjective health status to Model 1.
