# Supplementary material for: The relationship between poor sleep quality measured by the Pittsburgh Sleep Quality Index and smoking status according to sex and age: an analysis of the 2018 Korean Community Health Survey
Source: Epidemiol Health. 2022 Feb 14;44:e2022022. doi: 10.4178/epih.e2022022 (PMC9117098; doi:10.4178/epih.e2022022)
Supplement: Supplementary Material 2 — Unadjusted and adjusted odds ratios for poor sleep quality (PSQI > 5) according to smoking status (non-smoker subdivided into never-smoker and past smoker) by sex and age group [file epih-44-e2022022-suppl2.docx]

Supplementary Material 2. Unadjusted and adjusted odds ratios for poor sleep quality (PSQI > 5) according to smoking status (non-smoker subdivided into never-smoker and past smoker) by sex and age group

| Category | Subgroup | Male | | |  | Female | | |
| --- | --- | --- | --- | --- | --- | --- | --- | --- |
| Age |  | Crude OR  (95% CI) | Adjusted OR^1^  (95% CI) | Adjusted OR^2^  (95% CI) |  | Crude OR  (95% CI) | Adjusted OR^1^  (95% CI) | Adjusted OR^2^  (95% CI) |
| 19-35 | Never-smoker | Reference | Reference | Reference |  | Reference | Reference | Reference |
|  | Past smoker | 1.41  (1.25-1.58)* | 1.35  (1.20-1.52)* | 1.25  (1.10-1.41)* |  | 2.45  (2.04-2.95)* | 2.32  (1.93-2.80)* | 1.79  (1.47-2.17)* |
|  | Occasional smoker & Daily smoker (<1 pack per day) | 1.29  (1.18-1.40)* | 1.23  (1.13-1.35)* | 1.12  (1.02-1.23)* |  | 2.19  (1.86-2.58)* | 2.09  (1.76-2.48)* | 1.54  (1.28-1.85)* |
|  | Daily smoker (≥1 pack per day) | 1.70  (1.51-1.93)* | 1.53  (1.34-1.74)* | 1.21  (1.05-1.39)* |  | 3.53  (2.19-5.71)* | 3.41  (2.10-5.52)* | 1.59  (0.97-2.60) |
| 36-64 | Never-smoker | Reference | Reference | Reference |  | Reference | Reference | Reference |
|  | Past smoker | 1.20  (1.13-1.28)* | 1.12  (1.05-1.20)* | 1.09  (1.02-1.17)* |  | 1.67  (1.47-1.91)* | 1.56  (1.36-1.78)* | 1.25  (1.09-1.44)* |
|  | Occasional smoker & Daily smoker (<1 pack per day) | 1.26  (1.17-1.35)* | 1.20  (1.16-1.28)* | 1.07  (1.00-1.15) |  | 2.27  (2.02-2.56)* | 2.01  (1.78-2.27)* | 1.57  (1.38-1.79)* |
|  | Daily smoker (≥1 pack per day) | 1.48  (1.38-1.59)* | 1.33  (1.23-1.43)* | 1.12  (1.04-1.21)* |  | 2.56  (1.96-3.34)* | 2.10  (1.61-2.75)* | 1.50  (1.15-1.97)* |
| ≥65 | Never-smoker | Reference | Reference | Reference |  | Reference | Reference | Reference |
|  | Past smoker | 1.19  (1.10-1.29)* | 1.16  (1.07-1.26)* | 1.12  (1.03-1.21)* |  | 1.16  (0.96-1.41) | 1.11  (0.91-1.34) | 1.03  (0.85-1.25) |
|  | Occasional smoker & Daily smoker (<1 pack per day) | 1.25  (1.17-1.40)* | 1.20  (1.07-1.35)* | 1.11  (0.98-1.24) |  | 1.56  (1.23-1.99)* | 1.54  (1.21-1.95)* | 1.27  (0.99-1.62) |
|  | Daily smoker (≥1 pack per day) | 1.14  (0.99-1.31) | 1.14  (0.99-1.31) | 1.02  (0.88-1.19) |  | 1.52  (0.93-2.50) | 1.53  (0.93-2.51) | 1.06  (0.60-1.87) |

OR, odds ratio; CI, confidence interval

*p < 0.05

^1^ Adjusted for frequency of age, education level, employment, alcohol consumption, walking exercise, and history of hypertension or diabetes.

^2^ Additionally adjusted for perceived stress level, depressive mood, and subjective health status to Model 1.
